# The Influence of Pretherapeutic and Preoperative Sarcopenia on Short-Term Outcome after Esophagectomy

**DOI:** 10.3390/cancers12113409

**Published:** 2020-11-17

**Authors:** Johanna Grün, Lea Elfinger, Han Le, Christel Weiß, Mirko Otto, Christoph Reißfelder, Susanne Blank

**Affiliations:** 1 Department of Surgery, Universitäts Medizin Mannheim, Medical Faculty Mannheim, Heidelberg University, 68167 Mannheim, Germany; johanna.gruen@umm.de (J.G.); lea.elfinger@stud.uni-heidelberg.de (L.E.); Ngoc.Le@stud.uni-heidelberg.de (H.L.); mirko.otto@umm.de (M.O.); Christoph.reissfelder@umm.de (C.R.); 2 Department of Medical Statistics and Biomathematics, Medical Faculty Mannheim, Heidelberg University, 68167 Mannheim, Germany; christel.weiss@medma.uni-heidelberg.de

**Keywords:** skeletal muscle index, esophagectomy, nutritional status, sarcopenia

## Abstract

**Simple Summary:**

Although introducing minimally invasive surgery reduced postoperative morbidity after esophagectomy esophageal cancer still is a malignancy with poor prognosis. This study aimed to investigate whether preoperative sarcopenia has an influence on short-term postoperative outcome after esophagectomy in esophageal cancer patients. Our findings suggest that preoperative sarcopenia is no independent prognostic factor for postoperative outcome after esophagectomy but that patients’ nutritional status consists of more factors than only body mass index (BMI) and muscle mass. Prehabilitation and preoperative optimization of the patients’ nutritional status seems to be an important factor for short-term postoperative outcome after esophagectomy.

**Abstract:**

By introducing minimally invasive surgery the rate of postoperative morbidity in esophageal cancer patients could be reduced. But esophagectomy is still associated with a relevant risk of postoperative morbidity and mortality. Patients often present with nutritional deficiency and sarcopenia even at time of diagnosis. This study focuses on the influence of skeletal muscle index (SMI) on postoperative morbidity and mortality. Fifty-two patients were included in this study. SMI was measured using computer tomographic images at the time of diagnosis and before surgery. Then, SMI and different clinicopathological and demographic features were correlated with postoperative morbidity. There was no correlation between SMI before neoadjuvant therapy (*p* = 0.5365) nor before surgery (*p* = 0.3530) with the short-term postoperative outcome. Regarding cholesterol level before surgery there was a trend for a higher risk of complications with lower cholesterol levels (*p* = 0.0846). Our findings suggest that a low preoperative SMI does not necessarily predict a poor postoperative outcome in esophageal cancer patients after esophagectomy but that there are many factors that influence the nutritional status of cancer patients. To improve nutritional status, cancer patients at our clinic receive specialized nutritional counselling during neoadjuvant treatment as well as after surgery.

## 1. Introduction

Esophageal cancer is the eighth most common type of malignancy worldwide. As diseases like reflux and obesity are increasing worldwide, the incidence of Barrett’s esophagus as well as the incidence of esophageal adenocarcinomas have been increasing during the last decades. Although the outcome after curative multimodal therapy for esophageal cancer improved in the last decades, esophageal cancer still has a poor prognosis with a 10-year survival rate of approximately 16–17% and 5-year survival rate of approximately 20% [1,2].

By introducing minimally invasive procedures including the robot-assisted esophagectomy the rate of postoperative morbidity after esophagectomy could be reduced [3,4]. But esophagectomy is still associated with a relevant risk of postoperative morbidity, especially respiratory complications and anastomotic leakage including the risk of mediastinitis [5,6]. Thirty-day mortality after esophagectomy ranges between 1% and 3% and is mostly a result of postoperative complications. Postoperative complications affect short-term but also long-term survival as well as quality of life [6,7,8].

The Esophageal Complication Consensus Group presented an evaluation of 2704 patients with an overall complication rate of 59%. In an evaluation of 1057 total minimally invasive transthoracic esophagectomies, 56% of the patients developed at least one complication after surgery of which 26.9% were classified as Clavien Dindo grade III or more [6,9].

There are a number of studies identifying risk factors for a poor short-term outcome after surgical resection of esophageal carcinomas, such as high age, congestive heart failure, coronary artery disease, peripheral vascular disease, hypertension, body mass index <25 kg/m^2^, and insulin dependent diabetes [10,11].

It has also been shown that the patient’s nutritional status (including serum albumin, body mass index, muscle mass) plays an important role in determining patient outcome after surgery [12,13,14]. Patients with esophageal cancer often present with advanced disease and an impaired nutritional status due to dysphagia and cancer cachexia [15,16,17]. A good nutritional status before surgery has been proven to reduce the hypermetabolic response to surgery and to optimize wound and anastomotic healing and recovery [18,19,20].

Sarcopenia is defined by the European Working Group on Sarcopenia in Older People as low skeletal muscle mass and strength and is a risk factor for surgical patients in general [21,22]. It is also known as a possible risk factor for morbidity and poor prognosis after esophagectomy and could already be shown with a widespread prevalence, ranging from 16% to 79% before surgery [23,24,25,26].

A tool to measure skeletal muscle depletion is the skeletal muscle index (SMI). The skeletal muscle mass can be measured as cross-sectional area of the total skeletal muscle volume (cm^2^) at L3 in computertomographic images. The SMI is calculated as total skeletal muscle volume (cm^2^)/height^2^ (m^2^) [27,28]. It has been shown to be a prognostic factor independent from the body mass index (BMI) in oncologic patients [29].

Due to these findings the oncological patients at the Surgical Department of the University Hospital Mannheim are encouraged to take part in nutritional consulting at our clinic or at other specialized practices before and after surgery. Our clinic uses standardized protocols, guided by “Enhanced recovery after surgery“(ERAS) protocols, to improve postoperative outcome and length of hospital stay [30,31,32,33].

As a patient’s nutritional status before surgery seems to play a major role for short term outcome, the aim of our study was to investigate whether sarcopenia, at the time of diagnosis or before surgery as well as BMI and serum albumin levels are risk factors for postoperative mortality and morbidity after minimally-invasive or robot-assisted esophagectomy.

## 2. Results

52 patients were included in the study. The demographic and clinicopathological data of these patients are presented in Table 1. Of the 52 patients in one patient the tumor could not be resected.

Twenty-eight patients (54%) were seen by the nutritional expert at our department preoperatively. All patients were seen by the nutritional expert postoperatively. Shakes with high concentration of proteins were advised to all patients preoperatively. Ten patients (19.2%) needed intravenous nutrition preoperatively.

Mean albumin level was 35 ± 4.7 g/dL, mean cholesterol level (199.83 ± 48.18) mg/dL.

In 6 patients (11.5%) a totally minimal invasive approach was not possible, two patients had to be converted to open surgery (conversion rate 3.8%).

Mean duration of surgery was 445 min (range 303–770).

### 2.1. Skeletal Muscle Index

The skeletal muscle index (SMI) ranged from 29.7 to 62.6 cm^2^/m^2^ at time of diagnosis and from 31.9 to 62.5 cm^2^/m^2^ before surgery (mean values (47.7 ± 8.6 cm^2^/m^2^ and 42.1 ± 7.0 cm^2^/m^2^ respectively)).

Applying the cut-offs for sarcopenia used by Prado et al [34]. Of the patients, 54.3% were sarcopenic at time of diagnosis and 87.5% before surgery.

A moderate correlation was detected between BMI and SMI (r = 0.69855, *p* < 0.001, Pearson’s correlation coefficient).

Results regarding SMI, weight and BMI are summarized in Table 2. BMI at diagnosis is missing in 10 patients.

### 2.2. Short Term Postoperative Outcome

Short term postoperative outcome was defined as complications during hospital stay and complication associated mortality.

The median length of hospital stay was 17.5 days (range 6–114 days), the length of stay at the Intensive Care Unit ranged from one to 77 days (median 17.5 days).

One patient died during the first 30 days after surgery (30-day mortality 1.9%). The 90-day mortality in our cohort did not differ from this value because there were no other patients who died within 90 days after surgery.

Four patients died because of complications of surgery, three of those after 90 days after surgery (complication associated mortality 7.69%).

The general complication rate was 59.6% (31 patients in total). Twenty-six patients had surgical complications (50%), of those 16 patients suffered from anastomosis insufficiency (30.8% anastomosis insufficiency rate). Twenty-three patients suffered from medical complications (44.2%), including cardiac complications (21.2%) and pulmonary complications (32.7%).

There was no correlation between SMI before neoadjuvant therapy nor before surgery with short-term postoperative outcome (*p* = 0.5365, *p* = 0.3530 respectively, *t*-test).

The BMI also had no influence on postoperative morbidity: *p* = 0.4228 (BMI before diagnosis), *p* = 0.1673 (BMI before neoadjuvant treatment), *p* = 0.2810 (BMI before surgery), *t*-test. Regarding cholesterol level before surgery there was a trend for a higher risk of complications with lower cholesterol levels ((207 ± 55) mg/dL vs. (191 ± 38) mg/dL, *p* = 0.0846, *t*-test).

Other factors such as patient age (*p* = 0.4361, *t*-test), sex (*p* = 0.6872, Fisher’s exact test), history of cardiac disease (*p* = 0.8615, Chi-square-test), history of pulmonary disease (*p* = 1.0, Fisher’s exact test), tumor stage ((y)pT: *p* = 0.7241, (y)pN: *p* = 1.0, Cochran–Armitag-test), and preoperative albumin levels (*p* = 0.3747, *t*-test) had no statistically significant influence on short-term postoperative outcome. The duration of surgery however correlated with the incidence of complications (*p* = 0.0034, *t*-test) as patients who had longer times of surgery showed a higher risk for complications (466 vs. 392 min). Patients who had another type of cancer before developing esophageal cancer also suffered more often from complications (*p* = 0.0049, Chi-square-test).

## 3. Discussion

Our findings suggest that a low preoperative SMI does not necessarily predict a poor postoperative outcome in esophageal cancer patients after esophagectomy. Neither BMI before neoadjuvant therapy nor before surgery shows a correlation with postoperative outcome in our patients’ collective. Only one patient presented with a BMI <20 kg/m^2^. This might be due to patient selection. We included in our analysis only patients who were eligible for a thoracoabdominal surgical approach, which means that they were in a sufficient general status of health to tolerate one-lung-ventilation. We also checked all patients for nutritional deficiencies and sent them to a nutritional expert, which explains why the body weight difference between the time of diagnosis and the time of surgery is quite low and which could also explain the missing impact of BMI and SMI in our patients’ collective. Often it is during neoadjuvant treatment that patients lose weight and muscle mass [35]. In our patients’ collective the mean BMI before and after neoadjuvant treatment did not differ (25.87 and 25.34 kg/m^2^) and most of the patients (70.95%) did not have a significant reduction of SMI (70.95% had less than 5% reduction). Of the patients, formula increase of SMI during neoadjuvant treatment. This shows that an improvement in patients’ general status during neoadjuvant treatment is feasible.

As the number of patients in this cohort was relatively low it is also possible that in a greater cohort an effect of preoperative SMI on postoperative outcome might also be greater. Nevertheless, we could not see even a trend to significant correlations between SMI and complications and also there are other studies that showed similar results concerning this topic [36,37].

The level of cholesterol seems to be associated with the short-term postoperative outcome in our patients’ collective, which underlines the importance of the nutritional status. The results are not statistically significant but this could be due to the relatively low number of patients included.

Malnutrition in esophageal cancer patients is caused by dysphagia and a reduced food intake but also by cancer cachexia, which is induced by systemic inflammation. Cachexia leads to loss of body weight, body fat, and skeletal muscle mass. The molecular mechanisms of cachexia are still not fully understood, additionally there exists no consistent definition [38].

It should be mentioned that only one third of our patients had a squamous cell carcinoma (SCC). Patients with SCC normally present with a more advanced malnutrition than patients with an adenocarcinoma of the esophagus as malnutrition is often already present before diagnosis of the tumor. 

Different studies showed significant relations between sarcopenia and poor outcome after surgery such as the study by Elliot et al. that showed that sarcopenia is significantly associated with major complications after surgery or the study by Järvinen et al. that showed a correlation with worse overall survival [14,25,35,39,40,41].

Grotenhuis et al. and Siegal et al. on the other hand could not find any correlations between sarcopenia and postoperative complications or survival rates [36,37]. Nakashima et al. could find a significant correlation between sarcopenia and higher anastomotic leakage rates and also between sarcopenia and worse overall survival but these significant results were only present in a subgroup of patients older than 65 years [42].

The definition for sarcopenia is not consistent. Some studies define their own cut-offs. We used the most common cut-offs suggested by Prado et al [34], resulting in a rate of sarcopenia of 87.5% after neoadjuvant therapy. There is a huge variation in the prevalence of sarcopenia in esophageal cancer patients in the literature, varying from 16% to 75% [43].

Nevertheless, the latest meta-analysis by Papaconstantinou et al. showed a significant increase in overall morbidity, respiratory complications and anastomotic leaks in esophageal cancer patients with sarcopenia after esophagectomy. There were no statistically significant differences in overall mortality or Clavien–Dindo grade III or greater complications between patients with low and patients with normal SMI [44].

Although our findings contradict many studies that showed significant correlations between sarcopenia and higher complications or lower over-all survival there are other studies that support our findings.

There are some other important aspects of our patient cohort that should be illuminated. It has already been proven that prehabiliation and preoperative optimization of nutrition in cancer patients leads to better outcome and better quality of life after cancer surgery [45,46]. Nutrition goals include an adequate nutritional intake to prevent loss of muscle mass, modulate inflammation and the immune response, optimize glucose control, reduce the hypermetabolic response to surgery, and provide nutrients to optimize wound and anastomotic healing and recovery [18,19,20,47,48,49,50].

As mentioned above our cancer patients are provided with professional advice by specialized nutritional counsellors before and after surgery to improve their fitness and nutritional. This includes provision of protein shakes, dietary plans and even parenteral feeding if necessary.

It also has been shown that standardized protocols like ERAS protocols for surgical cancer patients lead to shorter in-hospital stays, decreased complications and longer overall survival [31,32,33,51]. Therefore, our clinic uses standardized pathways for esophageal cancer patients to improve postoperative outcomes of esophageal cancer patients.

This aspect of our preoperative treatment might be the reason for the absence of significant increase of postoperative morbidity or mortality in sarcopenic patients. One aspect that should be evaluated in further trials is, whether the outcome of esophageal cancer patients can be improved by introducing prehabilitation programs.

The presented study has some limitations. It is retrospective in nature. This increases the risk for systemic errors and selection bias. The number of patients included in this study (*n* = 52) is relatively low which may affect its statistical power, especially in regard to complication rates and specific complications.

## 4. Materials and Methods

### 4.1. Patient Selection and Study Design

The Surgical Department of the University Hospital Mannheim of Heidelberg University is a certified centre for esophageal surgery. A detailed clinico-pathologic database is prospectively maintained for all patients with esophageal cancer since January 2018 including data on short-term postoperative outcome. In this analysis we included patients having been operated between January 2018 and July 2019 in the Surgical Department of the University Hospital Mannheim of Heidelberg University. The local ethical committee “Ethikkomission II, University of Heidelberg” gave approval for the analysis (ethic code 2020-803R) and all patients gave written informed consent.

There were 52 patients who underwent surgery for treatment of esophageal cancer in whom CT scans were sufficient to determine the skeletal muscle index. Of the 52 patients, 48 underwent neoadjuvant chemotherapy before surgery (FLOT regimen).

The skeletal muscle index was measured using computer tomographic images at the time of diagnosis as well as before surgery.

Out of the 52 patients 13 did not have two usable computer tomographies either because they did not undergo neoadjuvant treatment or because imaging was not done in-hospital and was not sufficient for measurement of the SMI.

The skeletal muscle index (SMI) was calculated as the cross-sectional area of the total skeletal muscle volume (cm^2^) at L3.

At the level of the third lumbar vertebra (L3) we measured the area (cm^2^) of the left and right psoas major muscles, the side abdominal muscles, rectus abdominis muscles, erector spinae muscles, and quadratus lumborum muscles using “syngo.share view diagnostic” (Siemens Healthineers, Erlangen, Germany). Two adjacent axial images within the same series were selected, and total muscle cross-sectional area (cm^2^) at L3 was determined and averaged for each patient (1).

SMI *(cm^2^/m^2^) = Lean tissue area_L3_ (cm^2^)/height^2^ (m^2^)* (1)

Low SMI was defined as <52.4 cm^2^/m^2^ for male patients and as <38.5 cm^2^/m^2^ for female patients according to current literature [44].

### 4.2. Preoperative Treatment

All patients are seen by a surgeon specialized in upper GI surgery before admission to hospital. Education in nutrition as well as recommendation for daily physical activity and cessation of smoking is given during the consultation. Patients with nutritional deficiency are additionally sent to a nutritional consulting at the University Hospital Mannheim to compensate nutritional deficiencies before surgery.

Body weight, BMI, serum albumin levels, dysphagia, and subjective well-being are measured regularly by specialized nutritional consults. If the patients are suffering from dysphagia, weight loss or low BMI their nutritional status is treated by prescribing protein shakes, dietary plans and, if necessary parenteral feeding.

### 4.3. Neoadjvuant and Perioperative Therapy

Patients with locally advanced tumors (>cT2N0) receive neoadjuvant or perioperative treatment according to the FLOT protocol for adenocarcinomas and according to the CROSS protocol for squamous cell carcinomas [52].

After completion of preoperative treatment patients are reevaluated including a risk assessment and preoperative blood management.

### 4.4. Surgery

Surgery is performed by minimally invasive procedure, either laparoscopic and thoracoscopic approach or laparoscopic abdominal approach and robot-assisted thoracal approach. The decision for a robotic approach is taken due to availability of the DaVinci robot. Resection is done according to Ivor–Lewis procedure (right-thoracic approach). Lymphadenectomy is performed according to current guidelines including mediastinal lymphadenectomy and abdominal lymphadenectomy (D2 Lymphadenectomy). Reconstruction is done by gastric conduit and side-to-side esophagogastrostomy.

### 4.5. Postoperative Management

To ensure a standardized treatment of patients after surgery, the postoperative hospital management follows an in-house pathway which defines postoperative nutrition, mobilization, drainage management, laboratory tests, and dismissal after esophagectomy.

Directly after surgery patients stay in an Intermediate Care Unit (IMCU) for at least two days to ensure a balanced fluid management and to detect early postoperative complications as soon as possible.

Patients are encouraged to engage in light physical activity beginning on the day of surgery. Breathing exercises are started on the first postoperative day and should be done at least every hour for five to ten minutes.

Increased patient self-sufficiency in the general ward helps to improve mobilization and nutrition.

Nutritional counselling is performed during hospital treatment to determine nutritional deficiencies and to arrange nutritional support after the dismissal.

### 4.6. Postoperative Complications

Postoperative complications were investigated separately as surgical and medical complications as well as complications in total. To measure complication rates the Comprehensive Complication Index (CCI) was used as well as Clavien–Dindo classification.

The Clavien–Dindo classification is a commonly used tool with which usually the single most severe complication occurring in a patient during a given episode of care is reported [53].

The CCI is a relatively new tool to measure complications based on the Clavien–Dindo classification. In contrast to the Clavien–Dindo classification the CCI integrates all postoperative complications with their respective severities, on a scale ranging from 0 (no burden from complications) to 100 (death). The CCI is calculated by adding the weights of different complications (wC) and using a defined formula (2) [54].

CCI *= [√(wC_1_+wC_2_…+wC_x_)]/24.7* (2)

### 4.7. Statistical Analysis

For quantitative, approximately normally distributed variables, the mean and standard deviation have been calculated. Qualitative variables are given as absolute and relative frequencies. The median, together with range, are presented for skewed or ordinally scaled parameters. A Student’s *t*-test was used for comparing approximately normally distributed quantitative variables. The Cochran–Armitage test for trend was used to determine the influence of (y)pT and (y)pN categories on major postoperative complications. For qualitative variables, a χ^2^-test or Fisher exact test was performed, as appropriate. To determine a correlation between BMI and SMI the Pearson correlation coefficient was determined. All statistical tests for the comparison of 2 groups were two-tailed. In general, a test result was considered statistically significant if *p* < 0.05. All statistical analyses were performed using the SAS statistical analysis software, release 9.4 (SAS Institute Inc., Cary, NC, USA).

## 5. Conclusions

Our findings suggest that the nutritional status and physical fitness of esophageal cancer patients consist of more than BMI and skeletal muscle mass but that albumin levels, cardiac health and subjective well-being, as a consequence of adequate nutrition preoperatively, are important factors for a good postoperative outcome.

In the era of multimodal approach to esophageal cancer, treatment should consist not only of oncological treatment and surgery but also of a more systemic approach including patient education, physical exercise, and nutritional support. The period between diagnosis and surgery should be used to reduce cachexia and to better the general status of health.

Therefor prehabilitation programs as well as nutritional plans should be evaluated in clinical studies with the aim to give a consistent recommendation for patients with esophageal cancer [55].

## Figures and Tables

**Table 1:** Demographic and clinicopathological characteristics of patient cohort.

Clinicopathological/Demographic Features	Characteristics	Total (*n* = 52)
Sex	Female	7 (13.5%)
	Male	45 (86.5%)
Age (years)	Mean	67.4 ± 12.0
	<65 years	19 (36.5%)
	≥65 years	33 (63.5%)
Histopathology	SCC	17 (32.7%)
	AEG I	17 (32.7%)
	AEG II	17 (32.7%)
	AEG III	1 (1.9%)
(y)pT	0	7 (13.73)
	1	11 (21.6%)
	2	6 (11.8%)
	3	25 (49.0%)
	4	2 (3.9%)
(y)pN	0	28 (55.0%)
	1	12 (23.5%)
	2	5 (9.8%)
	3	6 (11.8%)
(y)pM	x	50 (96.1%)
	1	2 (3.9%)
R-status	R0	50 (96.2%)
	R1	2 (3.8%)
Lymph node resection (number)	Median	21
	Range	12–56
Lymph node ratio	Median	0.07
	Range	0–0.61
Previous diseases	Cardiac	28 (53.9%)
	Pulmonary	6 (11.5%)
	Diabetes	3 (5.8%)
	Other types of cancer	10 (19.23%)
Preoperative chemotherapy		48 (92.3%)
Type of Surgery	DaVinci-assisted	26 (50%)
	Laparoscopic	20 (38.5%)
	Open	6 (11.5%)
Clavien Dindo	0–3a	36 (69.2%)
	>3a	16 (30.8%)
CCI (Comprehensive Complication Index)	Median	26.2
	Range	0–100

**Table 2:** Skeletal muscle index (SMI), weight, and body mass index (BMI) at different time points.

SMI, BMI and Weight Reduction	Classification	Total (%)
SMI at Diagnosis	Mean (cm^2^/m^2^)	47.7 ± 8.6
	Range (cm^2^/m^2^)	29.7–62.6
SMI before surgery	Mean (cm^2^/m^2^)	42.1 ± 7.0
	Range (cm^2^/m^2^)	31.9–62.5
Reduction of SMI	Mean (cm^2^/m^2^)	3.3 ± 4.2
Reduction of SMI	0–5%	16 (51.6%)
	>5%	9 (29.0%)
	<0%	6 (19.4%)
Reduction of weight	0–5%	12 (29.3%)
	>5%	11 (26.8%)
	<0%	18 (44%)
BMI at diagnosis	Mean (kg/m^2^)	25.87 (±3.75)
	Range (kg/m^2^)	19.6–34.6
BMI before surgery	Mean (kg/m^2^)	25.34 (±3.9)
	Range(kg/m^2^)	18.37–34.94
BMI at diagnosis	<20 kg/m^2^	2 (5%)
	20–25 kg/m^2^	18 (45%)
	25–30 kg/m^2^	16 (40%)
	>30 kg/m^2^	4 (10%)
BMI before surgery	<20 kg/m^2^	2 (3.9%)
	20–25 kg/m^2^	25 (48.1%)
	25–30 kg/m^2^	17 (32.7%)
	>30 kg/m^2^	8 (15.4%)
